# Does Bipolar Disorder Get Worse at Geriatric Ages?

**DOI:** 10.1192/j.eurpsy.2023.841

**Published:** 2023-07-19

**Authors:** M. Dagtekin, H. Ertekin

**Affiliations:** psychiatry, Canakkale 18 Mart University, Çanakkale, Türkiye

## Abstract

**Introduction:**

Bipolar disorder is characterized with recurrent manic and depressive episodes with interepisodic remission periods. The course of illness including frequency and severity of mood episodes are the most evident changes at geriatric ages in bipolar disorders.

**Objectives:**

With this background, we aim to evaluate the clinical variables of bipolar patients older than 60 years and compare clinical variables before and after this age.

**Methods:**

Bipolar patients who applied to psychiatry outpatient unit in Çanakkale 18 Mart University Medical Faculty between the years of 2017-2022 were evaluated retrospectively. Patients over the age of 60 were included in the study. 47 out of 133 people over the age of 60 with bipolar disorder were not included in the study due to lack of information. Socio-demographic data of 85 patients recruited for the study, and clinical variables of the patients before and after the age of 60 were compared with Wilcoxon test. SPSS 26 version was used for statistical analysis and p<0.05 was considered as significance level.

**Results:**

When we evaluate the sociodemographic variables of the patients, we found that 61.2% (n=52) of the patients were female, mean age was 67.6± 6.3 years and mean duration of education was 7.2±4.6 years. Most of the patients (76.5%, n=65) was diagnosed with bipolar disorder type 1 (BP1) while nearly one four of them (24.7%) had a mood disorder history among their relatives. Median of the illness duration was 19.5 years (min:2, max:60), mean age of the first episode was 43.6±14.3 years and more than half had their first episode as depression (56.5%, n=48). When we compare the number of episodes, number and duration hospitalizations before and after the age of 60 years, we found that number of depressive (p=0.001,z:-3.3), (hypo)manic (p=0.001,z:-3.3), episodes and number of hospitalizations (p<0.001,z:-3.8), were lower at geriatric ages. However, there was no difference before and after the age of 60 years in terms of duration of hospitalization.

**Conclusions:**

Course of illness in bipolar disorder is highly variable and recurrence of mood episodes may increase with age (van der Markt A et al . Int J Geriatr Psychiatry. 2022;3;37(11), Dols A et al, The clinical course of late-life bipolar disorder, looking back and forward. 2017 Dec 11). However, in our study we found that number of depressive, (hypo)manic episodes and number of hospitalizations were lower at geriatric ages. This discrepancy may be related with sample selection and study design. Nevertheless, it should be taken into account for further studies. Besides, this is not a mirror image study and duration of follow-up periods were not considered for the statistical analysis. These are the additional limitation of our study. It is difficult to make further interpretations considering these limitations. Prospective follow-up studies with large sample size are required to better understand the course of bipolar disorder at geriatric ages.

**Disclosure of Interest:**

None Declared

